# Antibody dynamics in dogs submitted to different canine visceral leishmaniasis treatment protocols

**DOI:** 10.1590/S1984-29612025001

**Published:** 2025-01-13

**Authors:** Eliesse Pereira Costa, Gisele Vaz Aguirre Samoel, Gilneia da Rosa, Vanessa Osmari, Michelli Lopes de Souza, Luís Felipe Dias Lopes, Fernanda Silveira Flôres Vogel, Sônia de Avila Botton, Luís Antônio Sangioni

**Affiliations:** 1 Programa de Pós-graduação em Medicina Veterinária, Laboratório de Doenças Parasitárias, Departamento de Medicina Veterinária Preventiva, Universidade Federal de Santa Maria – UFSM, Santa Maria, RS, Brasil; 2 Departamento de Ciências Administrativas, Universidade Federal de Santa Maria – UFSM, Santa Maria, RS, Brasil; 3 Laboratório de Saúde Única, Departamento de Medicina Veterinária Preventiva, Universidade Federal de Santa Maria – UFSM, Santa Maria, RS, Brasil

**Keywords:** Infection, Leishmania, public health, zoonosis, Infecção, Leishmania, saúde pública, zoonoses

## Abstract

This study evaluated dynamics of antibodies in dogs treated for canine visceral leishmaniasis (CVL). Twenty-one dogs naturally infected by *Leishmania* spp. were grouped based on the treatment protocol: G1 (n=4) received allopurinol; G2 (n=10) allopurinol with miltefosine; and G3 (n=7) allopurinol, miltefosine and Leish-Tec® vaccine. The dogs were monitored monthly for a period of one year. To verify serum antibody titers, an indirect immunofluorescence reaction was performed. We found that dogs from G1 and G2 had lower clinical scores and antibody titers, when compared to the parameters evaluated in pre-treatment; however, clinical relapses were observed in three animals. In G3, clinical scores were lower than pre-treatment; however, they presented relatively stable antibody titers and no clinical relapse was observed. All animals submitted to the evaluated treatment protocols showed relative improvement in clinical signs. Furthermore, the immune response of animals must be considered, given the challenges of parasitic loads in infections. Therefore, it is necessary to complement the methods of clinical and therapeutic monitoring of dogs with CVL in order to establish the risk of transmissibility of the agent in infected and treated dogs.

## Resumo

Este estudo avaliou a dinâmica de anticorpos em cães tratados para leishmaniose visceral canina (LVC). Vinte e um cães, naturalmente infectados por *Leishmania* spp., foram agrupados com base no protocolo de tratamento: G1 (n=4) recebeu alopurinol; G2 (n=10) alopurinol e miltefosina; e G3 (n=7) alopurinol, miltefosina e vacina Leish-Tec®. Os cães foram monitorados mensalmente pelo período de um ano. Para verificar os títulos de anticorpos séricos, foi realizada a reação de imunofluorescência indireta. Observou-se que os cães do G1 e G2 apresentaram escores clínicos e títulos de anticorpos mais baixos, em comparação com os parâmetros avaliados no pré-tratamento; no entanto, recidivas clínicas foram observadas em três animais. No G3, os escores clínicos foram mais baixos do que no pré-tratamento; no entanto, apresentaram títulos de anticorpos relativamente estáveis e não foram observadas recidivas clínicas. Todos os animais, submetidos aos protocolos de tratamento avaliados, apresentaram melhora relativa dos sinais clínicos. Além disso, a resposta imune dos animais deve ser considerada, dados os desafios das cargas parasitárias nas infecções. Portanto, faz-se necessária a complementação dos métodos de acompanhamento clínico e terapêutico de cães com LVC, a fim de estabelecer o risco de transmissibilidade do agente em cães infectados e tratados.

## Introduction

Visceral leishmaniasis, a zoonosis that affects public health, is caused by protozoan species belonging to the genus *Leishmania*. They infect multiple hosts, with dogs serving as the primary urban reservoir of infection (Ratzlaff et al., 2023). Further, these parasites are spread by the vectors hematophagous sand flies (Diptera: Psychodidae: Phlebotominae) (Osmari et al., 2022). Visceral leishmaniasis is characterized by different clinical manifestations in humans and animals (Ratzlaff et al., 2023). Clinical implications of visceral leishmaniosis are variable, since infected animals often do not present with observable clinical signs or present with a wide spectrum of clinical signs, such as lymphadenomegaly, alopecia, weight loss, keratoconjunctivitis, and onychogryphosis, with varying degrees of severity, including death (Galán‐Relaño et al., 2022).

In Brazil, the products intended for human use or products that are not registered are prohibited for the treatment of canine visceral leishmaniasis (CVL) (Brasil, 2008). In 2016, miltefosine (Milteforan®), was authorized for the treatment of CVL (Brasil, 2016). A previous study showed that treatment with miltefosine alone reduced *Leishmania* spp. multiplication; however, the parasite was not completely removed from infected dogs. In addition, high levels of antibody production were associated with various clinical signs owing to the deposition of immune complexes in various organs and tissues, manly kidney, liver and skin (Proverbio et al., 2014). Therefore, miltefosine has been used in combination with other drugs, such as allopurinol, a xanthine oxidase inhibitor (Koutinas et al., 2001; Manna et al., 2009), or immunotherapy (Araújo & Gondim, 2020) for the treatment of CVL. However, the effectiveness of treatments over time has not been evaluated, nor has the reservoir status of these hosts through drug administration.

Thus, in the present study, we followed animals that were subjected to different treatment protocols for CVL, in order to evaluate the dynamics of antibodies associated with clinical staging, for a period of one year.

## Material and Methods

Dogs diagnosed with CVL and treated at the Hospital Veterinário Universitário of Universidade Federal de Santa Maria (HVU-UFSM) and private veterinary hospitals and clinics in Santa Maria, Rio Grande do Sul (Brazil), between August and September 2022 were included in the study and followed-up for 12 months during the treatment. Diagnosis of CVL was based on clinical manifestations, positive immunofluorescence antibody test (IFAT) for anti-*Leishmania* spp. serum antibody, and detection of *Leishmania* spp. in lymph node aspirate samples. Dogs with negative IFAT results or those that had received prior treatment with leishmanicidal or leishmaniostatic drugs were excluded from the study.

The three groups initially consisted of ten animals, however, only the animals that the owners agreed to in the commitment form were monitored. Therefore, twenty-one dogs *Leishmania* spp. infected were divided into the following three groups: group one (G1) contained four dogs that were treated with allopurinol; group two (G2) contained ten dogs that were treated with a combination of miltefosine and allopurinol, such that after 28 days of combination therapy, allopurinol was continued at the same dosage until the end of the study period; and group three (G3) contained seven dogs that were treated with a combination of miltefosine and allopurinol along with Leish-Tec^®^ vaccine regimen, which included three double doses of the vaccine, that were administered at an interval of 21 days between each dose, followed by a booster shot after 6 months (Araújo & Gondim, 2020). The therapeutic protocol for each dog was determined by a clinical veterinarian. One dog in G2 died due to complications from the disease 10 months post-treatment. Similarly, one dog in G3 died 7 months post-treatment, and one dog owner did not continue with the research group after 5 months post-treatment.

Dogs were clinically staged based on the presence of clinical signs that can be attributed to *Leishmania* spp. infection, according to the guidelines of Brasileish (2018). Blood samples (4 mL) were obtained from the animals by venipuncture of the cephalic vein or external jugular vein and stored in tubes without anticoagulants. The samples were centrifuged at 1000 × *g* for 5 min to obtain serum. Sera were aliquoted and stored at −20 °C until serological tests were performed.

Anti-*Leishmania* spp. antibodies were detected using an IFAT with an in-house protocol based on the protocols described previously by Lira (2005) and de Souza et al. (2022), with a titer of 40 as the cutoff (Brasil, 2014). For statistical analysis, IFAT anti-*Leishmania* antibody titers were graded on a scale of 0 to 9 (Manna et al., 2009). Blood samples were collected at multiple time points: before treatment and 1, 2, 3, 4, 5, 6, 7, 8, 9, 10, 11, and 12-months post-treatment.

Data are presented as the mean ± standard deviation (SD). Statistical analyses were performed using the nonparametric Kruskal–Wallis test. *P*-value less than 0.05 were considered statistically significant. All analyses were performed using BioEstat 5.0 statistical analysis software.

## Results and Discussion

The present study involved constant and systematic monitoring of twenty-one dogs with CVL over a period of 12 months. The mean clinical stages and IFAT scores of the study population are shown in Table 1 and 2, respectively. The pre-treatment scores of the three groups were based on the presence and severity of clinical signs associated with CVL. The average pre-treatment clinical scores for G1 (3), G2 (3), and G3 (2.8) were compared and were not statistically different *(p > 0,05)*. The interindividual variability (standard deviation, SD) in pre-treatment clinical scores of the groups was 1.15 for G1, 1.05 for G2 and 0.76 for G3. Moreover, the pre-treatment scores were also not significantly different *(p > 0,05)* from all other time points post-treatment (1 to 12 months) in all three treatment groups (Table 1).

**Table 1:** Clinical score changes in dogs in G1 (allopurinol), G2 (miltefosine+allopurinol) and G3 (miltefosine+allopurinol+Leish-Tec® vaccine) pre- and post-treatment, according to the guidelines of Brasileish (2018). Data are presented as mean and standard deviation.

TIME POINT (MONTHS POST-TREATMENT)	G1 (n = 4)	G2 (n = 10)^a^	G3 (n = 7)^b^	***P-value***
Pre-treatment	3*	3	2.8	0.7556
	(1.15)**	(1.05)	(0.76)	
1	2.3	2.8	2.2	0.2349
	(0.50)	(1.14)	(0.38)	
2	2.3	2.7	2.0	0.2032
	(0.50)	(1.06)	(0.58)	
3	2.3	2.4	2.2	0.7896
	(0.50)	(0.97)	(0.69)	
4	2.5	2.4	2.0	0.4689
	(0.58)	(0.97)	(0.58)	
5	2.3	2.3	2.2	0.9446
	(0.50)	(0.95)	(0.41)	
6	2.3	2.2	2.2	0.8060
	(0.50)	(0.92)	(0.41)	
7	2.3	2.1	2.0	0.8060
	(0.50)	(0.74)	(0.00)	
8	2.3	2.1	2.0	0.8060
	(0.50)	(0.74)	(0.00)	
9	2.3	2.2	2.0	0.8201
	(0.50)	(0.92)	(0.00)	
10	2.5	1.9	2.0	0.3747
	(0.58)	(0.78)	(0.00)	
11	2.0	1.9	2.0	0.2961
	(0.00)	(0.78)	(0.00)	
12	2.0	1.9	2.0	0.2961
	(0.00)	(0.78)	(0.00)	

* Mean;

** Standard Deviation;

a One dog in G2 died due to complications from the disease ten months post-treatment;

b One dog in G3 died seven months post-treatment and one dog owner did not continue with the research group five months post-treatment.

**Table 2:** IFAT scores of dogs in G1 (allopurinol), G2 (miltefosine+allopurinol), and G3 (miltefosine+allopurinol+Leish-Tec® vaccine) pre- and post- treatment. Data are presented as mean and standard deviation.

TIME POINT (MONTHS POST TREATMENT)	G1 (n = 4)	G2 (n = 10)^a^	G3 (n= 7)^b^	***P-value***
Pre-treatment	5.3*	5.9	3.8	0.1993
	(2.22)**	(2.73)	(2.15)	
1	6.3	6.4	4.0	0.0690
	(2.22)	(2.22)	(2.19)	
2	5.8	6.1	3.8	0.1161
	(0.96)	(2.13)	(2.37)	
3	5.0	5.3	4.5	0.4634
	(0.82)	(2.11)	(1.95)	
4	4.5	4.7	4.4	0.7699
	(1.00)	(2.11)	(2.64)	
5	4.0	5.0	4.0	0.3478
	(0.82)	(2.00)	(2.25)	
6	3.8	5.1	4.2	0.3191
	(0.50)	(2.02)	(2.66)	
7	3.5	4.9	3.8	0.2181
	(1.29)	(2.38)	(1.95)	
8	3.0	4.8	4.3	0.3593
	(0.82)	2.20	(3.58)	
9	2.7	4.5	4.0	0.5232
	(0.58)	(2.12)	(3.21)	
10	3.5	5.2	3.8	0.3173
	(1.29)	(2.22)	(3.49)	
11	3.8	4.6	3.8	0.5011
	(1.26)	(2.13)	(3.03)	
12	2.3	4.8	3.0	0.1422
	(1.26)	(2.28)	(2.70)	

* Mean;

** Standard Deviation;

a One dog in G2 died due to complications from the disease ten months post-treatment;

b One dog in G3 died seven months post-treatment and one dog owner did not continue with the research group five months post-treatment.

Based on the average IFAT scores pre-treatment and 12 months post-treatment, serum anti-*Leishmania* spp. antibody titers decreased progressively from 5.3 to 2.3 in G1 and from 5.9 to 4.8 in G2. However, serum anti-*Leishmania* spp. antibody titers were relatively stable in G3, with a pre-treatment titer of 3.8 and 12-months post-treatment titer of 3.0 (Table 2), consistent with that reported by Araújo & Gondim (2020). *Leishmania* spp. infect macrophages, and the Type 1 T helper (Th1) immune response is generally considered the main mechanism of parasite clearance during *Leishmania* spp. infection (Ikeogu et al., 2020), while Th2 immune response is correlated with disease progression and high parasitism (Leite et al., 2023).

**Figure 1:**
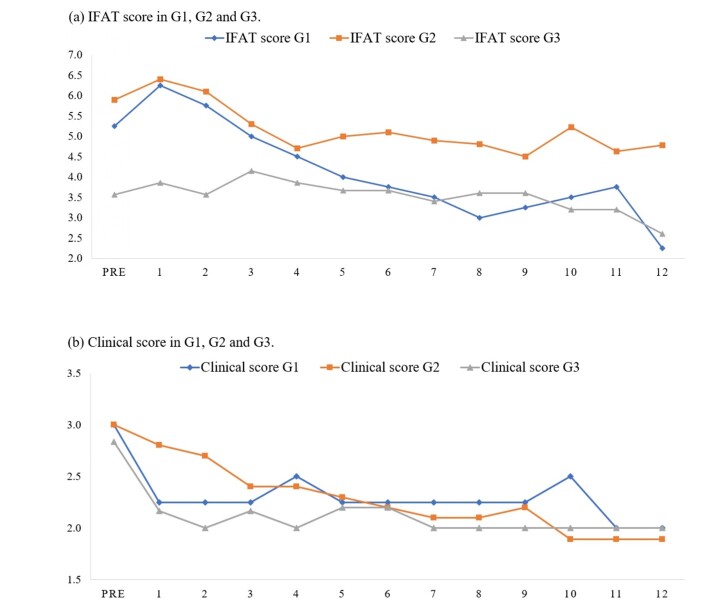
IFAT score (a) and Clinical score (b) in G1 (allopurinol), G2 (miltefosine+allopurinol) and G3 (miltefosine+allopurinol+Leish-Tec® vaccine) treated dogs during follow-up post-therapy, according to the guidelines of Brasileish (2018). Data are presented as mean.

Over the 12 months post-treatment follow-up period, 75% (3/4) of the dogs in G1 showed clinical improvement, with the disappearance of skin lesions; however, peripheral lymphadenopathy persisted. Despite strict adherence to treatment, 25% (1/4) of the dogs in G1 had remaining skin lesions with a reduction in serum anti-*Leishmania* spp. antibody titers reduced from a pre-treatment titer of 320 to a 12-month post-treatment titer of 40. In G2, 30% (3/10) of the dogs did not present with lymphadenopathy pre-treatment, and this was maintained throughout the course of treatment. In G2, 70% (7/10) of the dogs showed clinical improvement and disappearance of skin lesions, however one of the dogs had a clinical relapse 11 months post-treatment. Moreover, 20% (2/10) of the dogs had persistent skin lesions, of which one died 10 months post-treatment. It should be noted that 10% (1/10) never presented skin lesions. Regarding peripheral lymphadenopathy, 70% (7/10) dogs maintained this pathological change and 30% (3/10) never presented this condition. In G3, 86% (6/7) of the animals showed clinical improvement, with disappearance of skin lesions and a reduction in the size of lymph nodes; however, 14% (1/7) died 7 months post-treatment. In contrast, serum anti-*Leishmania* spp. antibody titers remained at relatively stable in G3 (Figure 1), which can be attributed to the use of immunotherapy and the stimulation of the Th1 immune response (Toepp et al., 2018). It was observed that, a reduction in serum anti-*Leishmania* spp. antibody titers were noted in both G1 and G2, 2 months post-treatment (Figure 1). Moreover, all therapeutic approaches resulted in a reduction in the average clinical scores (Figure 1).

In the present study, therapeutic failure was observed in one dog from G1 and another from G2, as the owners interrupted treatment before the end of the protocol. Consistent with this, clinical relapse and increased serum anti-*Leishmania* spp. antibody titers were observed in both dogs. The dog in G1 exhibited a progressive increase in serum anti-*Leishmania* spp. antibody titers from a pre-treatment titer of 160 to a titer of 640 in the subsequent months. Similarly, the dog in G2 showed an increase from a pre-treatment titer of 160 to a titer of 1,280 in the subsequent months. Negligence in the treatment of leishmaniasis worsens the animal's clinical condition and makes it infectious, which can cause the possibility of infections in other healthy dogs and other hosts, including humans (Travi et al., 2018). Additionally, clinical relapses were observed in one dog from G1 and two dogs from G2, even after adherence to the treatment protocol and in the absence of therapeutic failure. All dogs that relapsed exhibited increased clinical scores and serum anti-*Leishmania* spp. antibody titers, consistent with that reported by Manna et al. (2015). Recurrence of clinical signs in infected dogs represents a clinical challenge, not only because of the failure (Solano-Gallego et al., 2011) or exacerbated humoral immune response (Proverbio et al., 2014), but also possibly due to the resistance of this parasite to allopurinol (Yasur-Landau et al., 2016) and miltefosine (Gonçalves et al., 2024).

In the present study, the mean serum anti-*Leishmania* spp. antibody titer score was not related to the mean clinical score (*p > 0.05*); therefore, no causal relationship was observed in any of the groups. Consistent with our findings, Ayres et al. (2022) also reported no relationship between the results of serological tests and clinical improvement. In general, in the present study, clinical improvement was observed; however, some dogs exhibited worsening of clinical condition. This was noted in all three treatment groups. Clinical recurrence of CVL can be attributed to complex interactions between the host immune system and the parasite (Dias et al., 2020; Manna et al., 2006). Besides, clinical/therapeutic trials are important for clinical practice, nonetheless they have their limitations.

It should be noted that, due to the challenges of parasitic loads in infections, the immune response of animals must be considered in controlling the infection. It is noteworthy that the evaluation of antibodies in isolation does not constitute a good parameter for therapeutic evaluation.

## Conclusions

All animals submitted to the evaluated treatment protocols showed relative improvement in clinical signs. Furthermore, the immune response of animals must be considered, given the challenges of parasitic loads in infections. Therefore, it is necessary to complement the methods of clinical and therapeutic monitoring of dogs with CVL in order to establish the risk of transmissibility of the agent in infected and treated dogs.
